# A new framework for assessing subject-specific whole brain circulation and perfusion using MRI-based measurements and a multi-scale continuous flow model

**DOI:** 10.1371/journal.pcbi.1007073

**Published:** 2019-06-25

**Authors:** Erlend Hodneland, Erik Hanson, Ove Sævareid, Geir Nævdal, Arvid Lundervold, Veronika Šoltészová, Antonella Z. Munthe-Kaas, Andreas Deistung, Jürgen R. Reichenbach, Jan M. Nordbotten

**Affiliations:** 1 Norwegian Research Centre, Bergen, Norway; 2 Mohn Medical Imaging and Visualization Centre, Department of Radiology, Haukeland Universitetssykehus, Bergen, Norway; 3 Department of Mathematics, University of Bergen, Bergen, Norway; 4 Department of Biomedicine, University of Bergen, Bergen, Norway; 5 Medical Physics Group, Institute of Diagnostic and Interventional Radiology, Jena University Hospital - Friedrich Schiller University Jena, Germany; 6 Department of Neurology, Essen University Hospital, Essen, Germany; 7 Michael Stifel Center Jena for Data-driven and Simulation Science, Friedrich Schiller University, Jena, Germany; Stanford University, UNITED STATES

## Abstract

A large variety of severe medical conditions involve alterations in microvascular circulation. Hence, measurements or simulation of circulation and perfusion has considerable clinical value and can be used for diagnostics, evaluation of treatment efficacy, and for surgical planning. However, the accuracy of traditional tracer kinetic one-compartment models is limited due to scale dependency. As a remedy, we propose a scale invariant mathematical framework for simulating whole brain perfusion. The suggested framework is based on a segmentation of anatomical geometry down to imaging voxel resolution. Large vessels in the arterial and venous network are identified from time-of-flight (ToF) and quantitative susceptibility mapping (QSM). Macro-scale flow in the large-vessel-network is accurately modelled using the Hagen-Poiseuille equation, whereas capillary flow is treated as two-compartment porous media flow. Macro-scale flow is coupled with micro-scale flow by a spatially distributing support function in the terminal endings. Perfusion is defined as the transition of fluid from the arterial to the venous compartment. We demonstrate a whole brain simulation of tracer propagation on a realistic geometric model of the human brain, where the model comprises distinct areas of grey and white matter, as well as large vessels in the arterial and venous vascular network. Our proposed framework is an accurate and viable alternative to traditional compartment models, with high relevance for simulation of brain perfusion and also for restoration of field parameters in clinical brain perfusion applications.

## Author summary

An accurate simulation of blood-flow in the human brain can be used for improved diagnostics and assignment of personalized treatment regimes. However, current algorithms are limited to simulation of blood flow within tumours only, and in terms of parameter estimation, traditional compartment models have limited accuracy due to lack of spatial connectivity within the models. As a remedy, we propose a data-driven computational fluid dynamics model where the geometric domains for simulation are defined from state-of-the art MR acquisitions enabling a segmentation of large arteries and veins. In the capillary tissue we apply a two-compartment porous media model, where the perfusion is pressure-driven and is defined as the transition of blood from arterial to venous side. In addition, we propose a model for dealing with the intermediate scale problem where the vessels are undetectable and the flow does not adhere to requirements of porous media flow. For this scale, we propose a support function distributing the fluid in a nearby region around the vessel terminals. Combining these elements, we have developed a novel full human brain blood-flow simulator.

## Introduction

Applying traditional compartment models to in vivo hemodynamic measurements provides clinically valuable parameters in a wide range of medical conditions, e.g. Alzheimer disease [1], stroke [2, 3] or cancer [4, 5]. In these approaches, a pharmacokinetic compartment model is fitted to tracer evolution time curves from perfusion acquisitions to extract estimates of physiological parameters. The methodology is applied to entire organs, or regional- or voxel-wise, depending on the application.

In the case of tracer-based measurements of brain hemodynamics, cerebral blood flow (CBF, or perfusion), cerebral blood volume (CBV), and mean transit time (MTT) are commonly extracted parameters from one-compartment (1C) models. However, a fundamental drawback of these methods has been previously pointed out: determining CBF from traditional 1C models is scale dependent, hence the results depend on discretization level [6–8]. In [9] it was demonstrated that 1C models are prone to substantial errors when applied to smaller computational units connected in space instead of entire organs. This implies that measurements of perfusion on different discretization scales can provide considerably varying results depending on the choice of imaging device and post-processing software. The major reason for scale dependency of traditional 1C models is the lack of spatial connectivity in the model, hence allowing for repeated counts of the same fluid volume when applying the model to spatially connected units.

Recognition of the deficiencies of traditional compartment models has led to the development of multiscale, continuous blood flow models. Such models are highly relevant for improved understanding of the conditions affecting both global blood flow and microrheology in disease states, such as, e.g. cerebral aneurysms and sickle cell anemia [10]. Treatment of patients, such as by means of neurosurgery, may also benefit from individualized models that describe complex geometrical phenotypes [11]. Multiscale blood flow models may also contribute to a better understanding of angiogenesis and interstitial flow in simulated tumor microvascular networks, thus providing a more comprehensive and descriptive model for drug delivery [12–15].

A challenging topic within multiscale flow models is the precise mathematical formulation of perfusion within a continuous flow model. A model of perfusion should be in accordance with the physiological interpretation of perfusion being considered as a feeding arterial flow of oxygenated blood into the tissue or an organ. As a solution, we adopt a continuous flow model in which perfusion is regarded as the volume flux of oxygenated blood, which transits from arterial to the venous side in a two-compartment (2C) model [16–19]. This understanding of perfusion is both mathematically strict and physiologically sound.

The vascular system is a geometrically highly complex tubular network connecting vessels at different spatial scales. One particular challenge of whole brain simulation of perfusion is how to connect flow on the various scales, ranging from the carotid artery lumen with diameter close to 6 mm [20] down to capillaries with diameters of approximately 6 μm [21]. A suitable continuum model for flow simulations is expected to care for both tissue inhomogeneity and anisotropy, with the inconvenience of requiring a large number of unknown modelling parameters. One common approach is therefore to represent the vessels as an inexpensive 1D flow model coupled with a 3D continuum model for the brain tissue [19, 22–24]. The simple geometry of a 1D model reduces the number of required modelling parameters, while the vascular geometry can be observed in dedicated MR acquisitions. However, well-posedness and stability of the solution at the interface between the 1D and 3D model is challenging [22, 23]. In the current work, we address this problem by introducing a local flow distribution region where interface conditions are governed by a mass conserving, smooth support function, hence ensuring stability of the system.

## Methods

**Fig 1:**
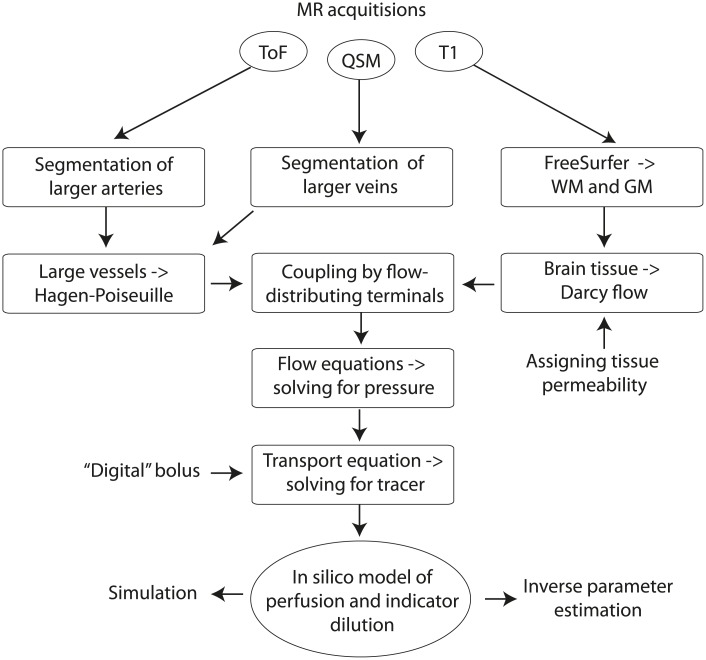
Workflow of algorithm for whole brain flow simulation. A FreeSurfer segmentation of the T1-weighted acquisition is used to define a brain mask from the union of white and grey matter. The ToF and QSM images were used to segment arteries and veins, respectively. Within the vascular network the flow is implemented according to the Hagen-Poiseuille equation. Flow in the brain is represented by Darcy flow, and coupled with Hagen-Poiseuille flow in the vascular tree using locally distributing source terminals. The resulting linear system is solved for the pressure. Flux is directly proportional to the pressure drop, and is used to compute tracer transport as a function of time. Finally, the workflow provides an in-silico model of whole brain perfusion and indicator dilution.

The workflow of our proposed method for whole brain simulation is schematically shown in Fig 1. Three dedicated MR acquisitions together with their appropriate data reconstruction and data post-processing are used to create the data-driven geometry: (i) A T1-weighted anatomical 3D data set used for segmenting white and grey matter, (ii) a time-of-flight (ToF) acquisition for identifying larger arteries, and (iii) a quantitative susceptibility map (QSM), which allows to extract larger veins. In the macro-scale network of arteries and veins we model the flow according to the Hagen-Poiseuille equation [25]. However, the brain also contains a large number of micro-scale capillaries, not recognizeable in space by any in vivo imaging device. So far, as a solution to this limitation, flow in capillaries is frequently modelled as porous media flow according to Darcy’s law [16, 17, 19]. We couple macro-scale Hagen-Poiseuille flow in large vessels with micro-scale Darcy flow in the capillaries by a set of locally distributing source terminals, hence providing a complete linear system, which is solved for node pressure values within the vascular network and voxel pressure values within the brain tissue. Finally, fluid flux obtained from the pressure gradient is used to simulate tracer transport, leading to an in-silico model of combined whole brain perfusion and tracer evolution. For the remaining, we make the assumption that our data represents healthy human brain tissue with an intact blood-brain barrier. Hence, no leakage of tracer into the extravascular space is expected, and one can exclusively model the tracer in the vascular space [26]. In the following, we carefully address individual processing steps.

### Data sets used for simulation

**Fig 2:**
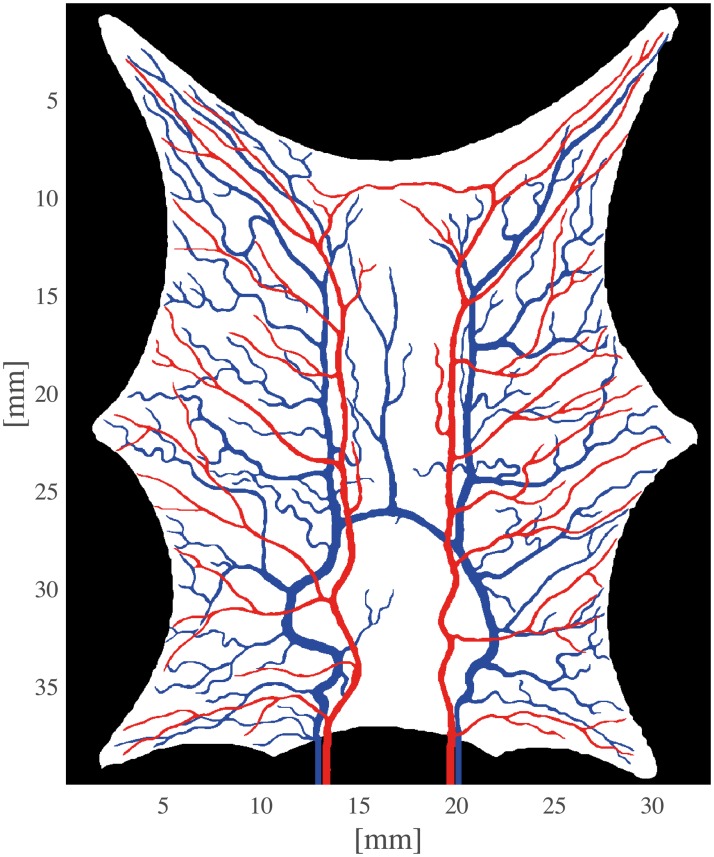
Geometry of a frog tongue with segmentation labels from dark to bright: Background (black), arterial network (red), venous network (blue), and tongue tissue (white). The sharp corners of the tongue tissue occur when the tongue is pinned down to the surface prior to imaging. The arterial input and venous outlet are in the bottom of the picture.

The purpose of the numerical simulations is twofold. First, by examples to illustrate working principles of our algorithm, and secondly, to demonstrate scale invariance. With this in mind, we have chosen the geometry of a frog tongue as example data [27]. This data set exhibits a realistic vascular geometry. Practically, the data set was scanned from a written source [27]. Preprocessing steps included semi-automatic segmentation of each of the vascular networks. Length of the tongue was measured to be 35 mm, and field of view (FOV) was set accordingly. As an approximation we consider the data to be almost two-dimensional. The tongue is also stretched between the nails pinning it to the surface, leading to a deformed geometry compared to a unprepared frog tongue. A visualization of the arterial and venous network, as well as the tongue tissue is shown in Fig 2. Input data can be found in Supporting Information S1–S3 Data. Furthermore, we used a full human brain for simulation of flow. Acquisition parameters and postprocessing steps of these data are described in the following.

#### MR acquisitions

Magnetic resonance (MR) imaging of a healthy male subject (age 26 years) was carried out at the Jena University Hospital (Jena, Germany) on a 3T MR system equipped with a 12 channel-phased-array receive head coil (Siemens Healthineers, Erlangen, Germany). Consent was not obtained because data were analyzed anonymously. A 3D, whole-head, T1-weighted MRI data set was collected by applying a magnetization-prepared rapid gradient echo (MP-RAGE) sequence (echo time TE = 3.24 ms, bandwidth BW = 200 Hz/px, repetition time TR = 2530 ms, inversion time TI = 1100 ms, flip angle FA = 9°, acquisition matrix = 320×240×224, voxel size = 0.8 mm×0.8 mm× 0.8 mm, acquisition time TA = 12:00 min:sec) for whole brain parcellation.

Image information about the cerebral arterial vessels was collected by performing a multi-slab time-of-flight (ToF) MR angiography (MRA) [28] with a single echo, 3D gradient-echo sequence (TE = 4.16 ms, BW = 180 Hz/px, TR = 23 ms, ramped FA = 20° [TONE pulse with 20° and TONE ratio 2:1] [29], acquisition matrix per slab = 448×346×64, voxel size = 0.49 mm×0.49 mm× 0.49 mm, TA = 32:57 min:sec). Signal saturation due to slow flowing arterial blood was reduced by acquiring six slabs with a slab overlapping factor of -20% [30]. Venous contamination in the ToF data was reduced by using additional venous saturation pulses.

To assess the cerebral venous vasculature, quantitative susceptibility mapping (QSM) was performed [31, 32]. For this purpose, data were acquired with a 3D, dual-echo gradient-echo sequence with flow compensation in all three spatial directions of the second echo (ToF-SWI sequence) [33]. The acquisition parameters included TE1 = 3.38 ms, BW1 = 272 Hz/px, TE2 = 24.7 ms, BW2 = 80 Hz/px, TR = 34 ms, acquisition matrix = 448×350×256, voxel size = 0.49 mm×0.49 mm×0.60 resulting in an acquisition time of 32:57 min:sec. The phase information of the second echo of the ToF-SWI sequence was used to compute magnetic susceptibility maps while applying sophisticated harmonic artefact reduction for phase data with variable radii (V-SHARP, 10 spherical kernels with radii ranging from 0.49 mm to 4.9 mm, regularization parameter [TSVD]: 0.05) [34, 35] for background field removal and homogeneity enabled incremental dipole inversion (HEIDI) [36] for field-to-susceptibility inversion. Finally, all three different contrasts were brought into a unified space with a common voxel size of 0.49 mm isotropic by linear registration, both the MP-RAGE and magnetic susceptibility maps to the ToF MRA data. Original input data can be found in Supporting Information S4–S7 Data. Shown pictures of the frog tongue and the human brain example were rescaled at double resolution and smoothed by anisotropic diffusion for visualization purposes.

#### Detection of brain tissue

FreeSurfer v6.0 (recon-all) was used to create a whole brain parcellation from the high-resolution T1-weighted dataset [37]. Masks of white and grey matter were extracted from the FreeSurfer parcellation. A brain mask was generated as the union of white and grey matter. These segmented data sets defined the geometry in the numerical example of whole brain perfusion. Porous media flow was applied only within the brain mask.

#### Detection of arterial and venous networks

**Fig 3:**
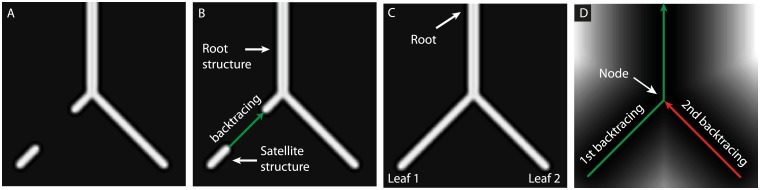
Automatic detection of vascular networks is divided into two consecutive workflows. (I) In the first part, (A) a connected, binary mask of the vascular network is generated from the input image (i.e. the ToF or the QSM in the case of a real application), here represented as a synthetic image demonstrating a small network of vessels. (B) Segmentation by adaptive thresholding creates a first approximation to the vascular network. However, due to local dropout in the signal, the segmented map also contains a satellite structure disconnected from the root structure. Computing the distance function around the root structure with the image itself as a speed function generates a favorable map which can be used for backtracing from the satellite structure to the root structure. This procedure generates a most probable path connecting these two structures (green path). (C) End points of the resulting, connected vascular network are either root points or leafs. (II) In the second part, from the connected network in (I), we identify leafs, root points, the skeleton, as well as network nodes. (D) Computing a distance function around the binary segmentation generates a map for a second backpropagation. A consecutive backpropagation from leaf 1 and 2 towards the root ensures a connected skeleton of the network. In addition, the procedure provides the nodes as the points of intersection of two paths of backpropagation, here indicated by the red arrow intersecting the green path.

The vessel detection procedure was split into two consecutive workflows, see Fig 3 for an overview. In the first workflow we create a connected, binary network. In a second workflow, we identify graph parameters, e.g. leafs, medial axes, nodes and edges. In the next, we describe the two workflows in more detail.

#### Detection of a connected, binary network

The ToF and QSM data were used for identifying arteries and veins, respectively (cfr. the workflow in Fig 3A–3C, where we have used an example image for demonstration purposes). Adaptive thresholding of each of these maps indicated most probable locations for vessels, resulting in a set of *N*_*R*_ disconnected components $\cup_{i=1}^{N_{R}}R_{i}$. The largest structure *R*_1_ is referred to as the root structure, while remaining structures are referred to as satellite structures (cfr. Fig 3B). Now, assume the components in $\cup_{i=1}^{N_{R}}R_{i}$ are sorted in descending order according to shortest Euclidean distance to the root structure. To overcome the problem that adaptive thresholding does not guarantee 3D connectivity, we solved the eikonal equation

$$\begin{array}{cc} & |\nabla T|=1/S,\;x\in \Omega \\ & T\left(x\right)=0,\;x\in R_{1} \end{array}$$ (1)

for the arrival time field T:Ω→R, *T* ≥ 0 and where Ω is the domain corresponding to the field-of-view (FOV). Eq (1) was solved by fast marching [38]. Backpropagation in the arrival time field provides the shortest path to the root from any point, given the velocity S:Ω→R, *S* ≥ 0. Using *S* equal to the ToF or QSM data facilitates a backpropagation along high intensity structures strongly associated with the path of arteries and veins, respectively. Backpropagation from the satellite structure *R*_2_ to the root structure *R*_1_ along the arrival time map *T*(*x*) provides the most probable path from *R*_2_ to *R*_1_ (cfr. Fig 3C). The region *R*_2_ is then assimilated into the root structure, *R*_1_ ← *R*_1_ ∪ *R*_2_, and the process of solving (1) is repeated for *i* = {2, 3, 4, …, *N*_*R*_}. Each backpropagation is applied until it reaches the growing root structure. Backpropagation ensures connectivity of every component *R*_*i*_ in the vessel network to the initial root structure. Backpropagation in the space between the connecting structures *R*_1_ and *R*_*i*_ was later combined with a dilatation of the connecting edge to produce tubular structures matching the average radius of (*R*_1_ + *R*_*i*_)/2 (cfr. Fig 3C). The resulting vascular network is referred to as the arterial or venous mask *R*_1_.

#### Identification of graph parameters

In the second part we identify leafs, medial axis, nodes and edges of the network (cfr. Fig 3D). To provide these parameters, let us first define a domain $\Omega_{B}^{CH}$ associated with the convex hull of the brain, as well as a binary image *B*_1_: Ω → {0, 1}, {*B*_1_(*x*) = 1 if *x* ∈ *R*_1_, else *B*_1_(*x*) = 0}. Then, we solve the boundary value problem

$$\begin{array}{llll} \left|\nabla T\right|= & \{\begin{array}{l} 1/\left(\text{dist}\left(B_{1}^{C}\left(x\right)\right)+\xi \right)\\ \text{inf} \end{array} & \begin{array}{l} \text{if}\\ \text{if} \end{array} & \begin{array}{l} x\in R_{1},\\ x\in \Omega /R_{1} \end{array}\\ T\left(x\right)= & 0 & \text{if} & x\in R_{1}\cap \partial \Omega_{ℬ}^{CH} \end{array}$$ (2)

for the arrival time *T*, where $B_{1}^{C}\left(x\right)$ is the complement of *B*_1_(*x*), *ξ* = 0.1 is a small number to avoid dividing by zero, and where dist() is the Euclidean distance function. Zero arrival time is set in the root points (i.e. the AIF points), defined as the intersection of the convex hull of the brain with the vessel network. Eq (2) provides a monotonically increasing map of arrival times along the arterial/venous network away from the root points. Leafs of the vascular network were identified from regional maxima (imregionalmax in MATLAB, cfr. Fig 3C), and backpropagation from the leafs towards the root leads to a set of spatially connected medial axis associated with the skeleton of the network. The path of backpropagation becomes the edges in the graph. Bifurcation points or nodes occur whenever a path of backpropagation intersects with a previous path of backpropagation, or with any of the root points (cfr. Fig 3D).

### Governing equations in the 3D domain

In the coupled model combining 1D flow in larger vessels with 3D Darcy flow in the brain, the majority of tissue is modelled as a porous medium where pressure driven flow is restricted by fluid mass balance and generic assumptions about the vascular microstructure of the arterioles, venules and the capillary system. In order to describe perfusion mathematically, we work under the assumption of two parallel 3D systems (or compartments), one accounting for arterial and one accounting for venous flow. The perfusion is interpreted as the delivery of oxygenated blood from the first to the latter compartment. Further details regarding the 3D model are given below.

#### Fluid mass balance

Let us now define a brain mask $\Omega_{B}$ as the union of grey and white matter. Fluid mass balance is ensured by the continuity equation, expressed in global form as

$$\begin{array}{c} \frac{d}{dt}\int_{\Omega_{i}}\phi \rho dx+\int_{\partial \Omega_{i}}\rho \left(u\cdot n\right)dA=\int_{\Omega_{i}}\tilde{Q}dx \end{array}$$ (3)

for a geometric control volume Ω_*i*_ with boundaries ∂Ω_*i*_. In (3), *n* is the outer unit normal vector of ∂Ω_*i*_, $u:\Omega_{B}\times T\to R^{3}$ is the flow per unit area (i.e. flux), [m^3^ s^−1^ m^−2^], $\rho :\Omega_{B}\times T\to R$ is the fluid density [kg m^−3^], $\phi :\Omega_{B}\times T\to R$ is the porosity [-], and $\tilde{Q}:\Omega_{B}\times T\to R$ is a fluid source term [kg s^−1^ m^−3^]. In geoscience, the parameter 0 ≤ *ϕ* ≤ 1 is known as the porosity, and in the field of neuroimaging it is known as cerebral blood volume (CBV). Eq (3) must be valid for every geometric control volume Ω_*i*_, hence, by the divergence theorem

$$\begin{array}{c} \frac{\partial }{\partial t}\left(\phi \rho \right)+\nabla \cdot \left(\rho u\right)=\tilde{Q}. \end{array}$$ (4)

For an incompressible fluid and for a situation of constant fluid density, (4) is equivalent with

$$\begin{array}{c} \nabla \cdot u=Q \end{array}$$ (5)

where $Q=\tilde{Q}/\rho$ has units [m^3^ s^−1^ m^−3^].

#### Flow equations in the brain

Assuming a low velocity flow within the capillary brain tissue according to Darcy’s law, provides the relation

$$\begin{array}{c} u=-\frac{k}{\mu }\nabla p \end{array}$$ (6)

between the flux $u:\Omega_{B}\to R^{3}$ [m^3^ s^−1^ m^−3^] and the pressure $p:\Omega_{B}\to R$ [Pa] when neglecting the gravitational acceleration [16, 17, 19, 39]. The flux *u* is also known as the Darcy velocity, and is related to the fluid velocity by the porosity since only a fraction of the geometric volume is available to flow. The viscosity *μ* is assumed to be constant everywhere, and $k:\Omega_{B}\to R$ [mm^2^] is the vascular permeability, in this work assumed to be isotropic.

Now, consider two fluid compartments, the arterial and venous compartment, where we employ an index *β* indicating the compartment i.e. *β* ∈ {*a* = arterial, *v* = venous}. We allow each compartment to possess heterogenous porosity *ϕ*_*β*_(*x*) and vascular permeability *k*_*β*_(*x*). However, in the current work, we assign regionally constant parameter values of porosity and vascular permeability within each compartment due to lacking prior information of regional variability. Furthermore, let the perfusion $P:\Omega_{B}\to R$ [m^3^ s^−1^ m^−3^] be the volume flux between the two compartments, hence understanding perfusion as the transition rate of blood from oxygenated to deoxygenated state [16–19]. Vascular flow is mainly pressure driven, and a legitimate model for perfusion is linearly proportional to the pressure difference between the arterial and venous compartment,

$$\begin{array}{c} P=\alpha (p_{a}-p_{v}) \end{array}$$ (7)

with a perfusion proportionality factor *α* = *α*(*x*) [m s kg^−1^]. The parameter *α* is assumed to reflect anatomical factors affecting the tissue’s ability to facilitate perfusion, e.g. capillary density and microstructural organization, and can later be refined to separate the various factors. Combining mass conservation (5) with porous media flow (6) for each of the two compartments while coupling the compartments with the perfusion (7) yields a set of partial differential equations in the pressure fields *p*_*a*_ and *p*_*v*_

$$\begin{array}{ccc} -\nabla \cdot (\frac{k_{a}\left(x\right)}{\mu }\nabla p_{a}\left(x\right)) & =\sum_{k\in T_{a}^{I}}Q_{a,k}^{\epsilon }\left(x\right)-P\left(x\right)\; & x\in \Omega_{B}\\ -\nabla \cdot (\frac{k_{v}\left(x\right)}{\mu }\nabla p_{v}\left(x\right)) & =\sum_{k\in T_{v}^{I}}Q_{v,k}^{\epsilon }\left(x\right)+P\left(x\right)\; & x\in \Omega_{B}\\ u_{\beta }\left(x\right)\cdot n_{\beta }\left(x\right) & =0 & \;\beta =\left\{a,v\right\},\;x\in \partial \Omega_{B} \end{array}$$ (8)

where *n*_*β*_ is the outer normal vector of the boundaries $\partial \Omega_{B}$ of $\Omega_{B}$. No-flow Neumann boundary conditions for the flux *u* ∝ ∇*p* are imposed for $x\in \partial \Omega_{B}$. The parameter *ϵ* is a local radius around *x* where $Q_{a,k}^{\epsilon }\left(x\right)$ has support, and will later be explained in more detail. Right hand side terms $Q_{\beta ,k}^{\epsilon }=Q_{\beta ,k}^{\epsilon }\left(x\right)$ [m^3^ s^−1^ m^−3^] are volumetric sink or source terms. The perfusion *P* becomes a sink term for the arterial compartment and a source term in the venous compartment.

### Governing equations in the vascular network

**Fig 4:**
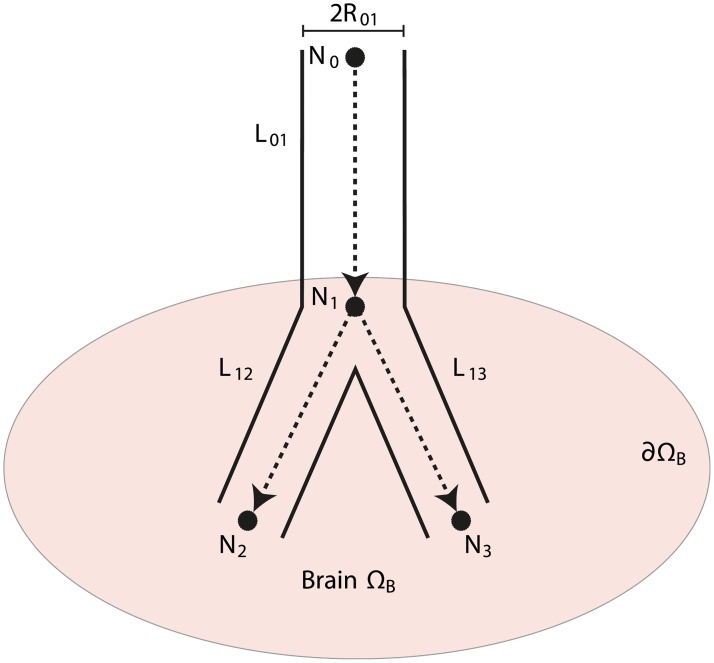
Illustration of an arterial network with nodes *N*_*i*_, *i* = {0, … 3} and connecting edges. Each edge has an associated length *L*_*jk*_, radius *R*_*jk*_ and medial axis (dashed lines). In the current example, *N*_0_, *N*_2_, *N*_3_ are terminal nodes, while *N*_0_ is also a root terminal mediating incoming fluid into the vascular network. Node *N*_1_ is an interior node. Brain tissue $\Omega_{B}$ is shown as a filled ellipsoid, and arrowheads indicate direction of flow. For the sake of illustration, only the arterial network is shown, but a similar, fluid collecting venous network is also present in the model.

For simplicity, we currently omit the index *β* = {*a*, *v*} and consider either the arterial or the venous vascular network consisting of nodes *N*_*i*_ and edges *E*_*jk*_. Each node *N*_*i*_ has an associated position ${\tilde{x}}_{i}$ and pressure ${\tilde{p}}_{i}$ [Pa]. An edge *E*_*jk*_ is the connection between the pair of nodes (*N*_*j*_, *N*_*k*_), and is associated with a tubular length *L*_*jk*_ [m] and a constant tubular radius *R*_*jk*_ [m]. The length *L*_*jk*_ is a geodesic distance measured along the tubular medial axis, and *R*_*jk*_ is computed as the average tubular radius along the structure *E*_*jk*_. Each edge *E*_*jk*_ mediates an absolute flow ${\tilde{q}}_{jk}$ [m^3^ s^−1^] from node *N*_*j*_ to node *N*_*k*_. Algorithmically, the network is represented as a connectivity matrix of an undirected graph. A schematic illustration of a vascular network with proper notation is shown in Fig 4.

Each node *N*_*i*_ is connected to a set of neighboring nodes $N(N_{i})$. A terminal node is defined as any node with only one neighbour, i.e. $N^{T}≔\{i:|N\left(N_{i}\right)\left|=1\right\}$ for the cardinality |⋅|, while interior nodes are nodes with more than one neighbour, $N^{I}≔\{i:|N\left(N_{i}\right)\left|>1\right\}$. We further split the terminal nodes into root terminals and interior terminals. Root terminals *N*^*R*^ are pressurized terminal nodes with imposed Dirichlet boundary conditions. Algorithmically, the root terminals are the intersection points of the large vessels with the brain-background interphase $\partial \Omega_{B}$, $N^{R}=\left\{i:{\tilde{x}}_{i}\in \partial \Omega_{B}\right\}$. Interior terminals are terminal nodes placed within the domain, mediating flow from/to the vascular tree into the 3D domain. The flow in the interior terminals is a Neumann boundary condition for the microvascular flow in the 3D domain. For the remaining, we refer to interior terminals as terminals. Finally, the set of all nodes is the union of interior nodes, terminals and root terminals, *N*^*I*^ ∪ *N*^*T*^ ∪ *N*^*R*^.

For modelling of flow through larger vessels we approximate the vessels as straight tubes of constant, circular cross-sections. We also assume laminar flow of an incompressible, Newtonian fluid. The assumption of laminar flow is supported by Reynold numbers < 200 for any of the middle cerebral arteries and penetrating arterioles [40]. Under these assumptions, the vascular flow in larger vessels can be modelled using the Hagen-Poiseuille equation, relating a pressure drop $\Delta {\tilde{p}}_{jk}≔{\tilde{p}}_{j}-{\tilde{p}}_{k}$ of an incompressible fluid of viscosity *μ* [Pa ⋅ s] through *E*_*jk*_ to the flow ${\tilde{q}}_{jk}$ between two nodes *N*_*j*_ and *N*_*k*_ [41],

$$\begin{array}{c} \Delta {\tilde{p}}_{jk}=\frac{8\mu L_{jk}{\tilde{q}}_{jk}}{\pi R_{jk}^{4}}. \end{array}$$ (9)

Fluid mass balance must be ensured for each interior node

$$\begin{array}{c} \sum_{j\in N(N_{k})}{\tilde{q}}_{jk}=0,\;k\in N^{I}. \end{array}$$ (10)

Denoting $\kappa_{jk}≔\pi R_{jk}^{4}/8\mu L_{jk}$, and combining (9) with (10) yields the relation

$$\begin{array}{c} \sum_{j\in N(N_{k})}\kappa_{jk}\left({\tilde{p}}_{j}-{\tilde{p}}_{k}\right)=0,\;k\in N^{I} \end{array}$$ (11)

for the fluid mass balance of each interior node. Within the arterial and networks we assume full porosity, hence collapsing into one-compartment flow. The root terminals represent the outer boundaries of the complete flow system, and each of the root terminals are therefore assigned a user-provided Dirichlet boundary condition ${\tilde{p}}_{0}$

$$\begin{array}{c} {\tilde{p}}_{k}={\tilde{p}}_{0}\;\forall \;k\in N^{R}. \end{array}$$ (12)

From the definition of a terminal node, terminal nodes have only one neighbour, i.e. only one edge connection, and the flow in each of the terminal nodes can be expressed as

$$\begin{array}{c} \tilde{q_{k}}=\kappa_{jk}\left({\tilde{p}}_{j}-{\tilde{p}}_{k}\right),\;j\in N\left(N_{k}\right)\;\forall \;k\in N^{I}\; \end{array}$$ (13)

providing Neumann boundary conditions of flow continuity between the arterial/venous network and the brain. This topic is further addressed in the next section.

### Distributing flow from the terminals

The intersection point connecting the flow in the 1D tubular network with the flow in the generic 3D tissue yields a singularity both in terms of physics and numerics. Physically, there is a modelling gap between the explicit, macro scale representation of arterial/veinal flow and the micro circulation in the 3D domain. In our model, we fill this gap using a volumetric source term *Q*^*ϵ*^ [m^3^ s^−1^ m^−3^]. For *ϵ* = 0 this is essentially a Dirac measure

$$\begin{array}{c} Q^{0}\left(x\right)=\int_{\Omega }Q\left(y\right)\delta \left(x-y\right)dy \end{array}$$ (14)

where all fluid is distributed within an infinitely small point. However, assume that the blood from the terminals is distributed along a fine scale network which is not visible at the imaging resolution. The idea is to replace the Dirac measure, which is not physically sound, with a more realistic model of the source region with a characteristic radius *ϵ*. To this end, define the support function

$$\begin{array}{c} \eta^{\epsilon }\left(x\right)≔\frac{1}{\epsilon^{n}}\eta (\frac{x}{\epsilon }) \end{array}$$ (15)

where the shape function *η* is any positive and continuously differentiable function satisfying $\int_{\Omega_{B}}\eta dx=1$. In principle, the shape function *η* should reflect the structure of the sub-resolution arterial and venous trees, but due to the lack of such data we adopt the generic choice

$$\begin{array}{c} \eta \left(x\right)≔\{\begin{array}{ccc} & C\;\text{exp}\;(\frac{1}{{\left|x\right|}^{2}-1}) & \;\text{if}\;|x[<1,\\ & 0 & \;\text{if}\;|x|\geq 1. \end{array} \end{array}$$ (16)

An appropriate expression for the source terms then becomes

$$\begin{array}{c} Q^{\epsilon }\left(x\right)=\int_{\Omega }Q\left(y\right)\eta^{\epsilon }\left(x-y\right)dy. \end{array}$$ (17)

Due to the properties that both the Dirac delta distribution and the source distribution integrate to unity, we note that the total integral over (14) and (17) remains the same and global mass balance is ensured from the terminals. Moreover, (17) converges to (14) in the case where *ϵ* → 0, justifying the notation *Q*^0^ in (14). For the remaining, adopt the notation of *β* indicating characteristics of the arterial (*β* = *a*) or venous (*β* = *v*) tree, e.g. single nodes *N*_*k*_ → *N*_*β*,*k*_ or the set of nodes $N^{j}\to N_{\beta }^{j},j\in \left\{I,T,R\right\}$. Considering the volumetric source/sink terms $Q_{\beta ,k}^{\epsilon }$ in (8), the total flow contribution (17) from terminal *k* can be approximated as

$$\begin{array}{c} Q_{\beta ,k}^{\epsilon }\left(x\right)=Q_{\beta ,k}\eta_{\beta ,k}^{\epsilon }\left(x-{\tilde{x}}_{k}\right)\left|N_{\beta ,k}\right|. \end{array}$$ (18)

where |*N*_*β*,*k*_| is the node volume. The volumetric flow *Q*_*β*,*k*_ through terminal *k* relates to the absolute flow ${\tilde{q}}_{\beta ,k}$ by

$$\begin{array}{c} Q_{\beta ,k}={\tilde{q}}_{\beta ,k}/\left|N_{\beta ,k}\right|, \end{array}$$ (19)

thus providing the relation

$$\begin{array}{c} Q_{\beta ,k}^{\epsilon }\left(x\right)={\tilde{q}}_{\beta ,k}\eta_{\beta ,k}^{\epsilon }\left(x-x_{k}\right). \end{array}$$ (20)

### Continuity in pressure

In the flow-distribution region around each interior terminal |*x* − *x*_*k*_| < *ϵ* we require that the pressure drop between a terminal node *N*_*β*,*k*_ and the surrounding brain tissue satisfying |*x* − *x*_*k*_| < *ϵ* scales with the terminal flow up to a user-provided constant *γ*_*β*_

$$\begin{array}{cc} {\tilde{q}}_{\beta ,k}=\frac{\gamma_{\beta }}{\mu }({\tilde{p}}_{\beta ,k}-\int_{\Omega }\eta_{\beta ,k}^{\epsilon }\left(x-x_{k}\right)p_{\beta }dx)\;\; & \forall \;k\in N_{\beta }^{I}. \end{array}$$ (21)

The coefficient *γ*_*β*_ has the interpretation of an effective resistance in the unresolved network extending from the terminal node. A higher value of *γ*_*β*_ will enforce a lower pressure drop between the vascular tree and the microvascular model, and vice versa. This closes the coupled modelling system, where the complete flow model is formed from (7), (8), (11), (12), (13), (20), (21).

### Generating maps of the perfusion proportionality factor *α*(*x*)

For the real brain application, voxelwise maps of the perfusion proportionality factor *α* (7) were generated with higher values in grey matter than in white matter [42], ensuring a physiologically reasonable map. We illustrate our approach by applying the method also to simulate flow in a frog tongue. In this specific example there is no grey or white matter, and we use *α* constant everywhere.

### Tracer mass balance and indicator dilution

The equations in the preceding sections describe blood propagation from the arterial to the venous side of the brain vascularity. In order to simulate a real contrast enhanced MR acquisition we also introduce a model for transport of a tracer in the bloodstream. All quantities are assumed to be in SI units, and later converted to more appropriate units for presentation whenever needed. Observable or volumetric tracer concentration *C*(*x*, *t*) [mol/m^3^] is a linear function of the fractional volumetric tracer concentrations *C*_*β*_(*x*, *t*) for each of the compartments

$$\begin{array}{c} C\left(x,t\right)=C_{a}\left(x,t\right)+C_{v}\left(x,t\right). \end{array}$$ (22)

Furthermore, tracer distribution volume is different from a geometric volume whenever *ϕ*_*β*_ < 1, leading to the relation

$$\begin{array}{c} C_{\beta }\left(x,t\right)=\phi_{\beta }\left(x\right)c_{\beta ,b}\left(x,t\right) \end{array}$$ (23)

connecting blood tracer concentration *c*_*β*,*b*_(*x*, *t*) [mol/m^3^] to volumetric tracer concentration *C*_*β*_(*x*, *t*).

The following criteria are assumed to hold: The tracer is homogeneously distributed in the fluid within a small distribution volume Ω_*i*_ (i.e. a voxel or a node), all physiological and structural parameters are stationary within the time of acquisition, and tracer transport by diffusion is not considered. Under these assumptions, the influx of tracer into Ω_*i*_ is determined by the product of fluid tracer concentration *c*_*β*_(*x*, *t*) and flux *u*_*β*_(*x*)

$$\begin{array}{c} -\int_{\partial \Omega_{i}}c_{\beta }\left(u_{\beta }\cdot n\right)dA \end{array}$$ (24)

where *n* is the outward pointing surface normal of Ω_*i*_. The rate of change of tracer within the control volume yields

$$\begin{array}{c} \frac{d}{dt}\int_{\Omega_{i}}C_{\beta }\left(x,t\right)dx. \end{array}$$ (25)

In addition, one must account for volumetric source terms. Combining (24) with (25) and allowing for perfusion and inflow and outflow of tracer from/to all interior terminals, an upstream finite volume model for tracer mass balance can be phrased as

$$\begin{array}{ccc} \int_{\Omega_{i}}\phi_{a}\frac{\partial c_{a}}{\partial t}dx & =-\int_{\partial \Omega_{i}}c_{a}\left(u_{a}\cdot n\right)dA-\int_{\Omega_{i}}c^{\prime \prime }Pdx+\sum_{k\in N_{a}^{I}}\int_{\Omega_{i}}c_{a}^{\prime }{\tilde{q}}_{a,k}\eta_{a,k}^{\epsilon }\left(x-x_{k}\right)dx\; & x\in \Omega_{B}\\ \text{for}\;c_{a}^{\prime } & =\{\begin{array}{ccc} & {\tilde{c}}_{a,k}\left(t\right) & \;\text{if}\;{\tilde{q}}_{a,k}\geq 0\;\text{(Node}\;\text{is}\;\text{upstream)}\\ & c_{a}\left(x,t\right) & \;\text{if}\;{\tilde{q}}_{a,k}<0\;\text{(Brain}\;\text{tissue}\;\text{is}\;\text{upstream)} \end{array}\\ \int_{\Omega_{i}}\phi_{v}\frac{\partial c_{v}}{\partial t}dx & =-\int_{\partial \Omega_{i}}c_{v}\left(u_{v}\cdot n\right)dA+\int_{\Omega_{i}}c^{\prime \prime }Pdx+\sum_{k\in N_{v}^{I}}\int_{\Omega_{i}}c_{v}^{\prime }{\tilde{q}}_{v,k}\eta_{v,k}^{\epsilon }\left(x-x_{k}\right)dx\; & x\in \Omega_{B}\\ \text{for}\;c_{v}^{\prime } & =\{\begin{array}{ccc} & c_{v}\left(x,t\right) & \;\text{if}\;{\tilde{q}}_{v,k}<0\;\text{(Brain}\;\text{tissue}\;\text{is}\;\text{upstream)}\\ & {\tilde{c}}_{v,k}\left(t\right) & \;\text{if}\;{\tilde{q}}_{v,k}\geq 0\;\text{(Node}\;\text{is}\;\text{upstream)} \end{array}\\ \;\text{and}\;\text{where}\;c^{\prime \prime } & =\{\begin{array}{ccc} & c_{a}\left(x,t\right) & \;\text{if}\;p_{a}\geq p_{v}\\ & c_{v}\left(x,t\right) & \;\text{if}\;p_{a}<p_{v} \end{array}. \end{array}$$ (26)

For the edges *E*_*β*,*jk*_ we construct a finer discretization in order to facilitate graded tracer concentration along the edges. Hence, split each edge *E*_*β*,*jk*_ into *n*_*β*,*jk*_ subsegments associated with medial axis voxels, and assign every remaining voxel in the edge to the closest medial axis point, leading to disc-like discretization volumes *E*_*β*,*jk*,*i*_ referring to subsegment *i* within edge *E*_*β*,*jk*_. Also, assume the order of subsegments is downstream with increasing index *i*. In particular, $c_{\beta ,jk,n_{\beta ,jk}}$ refers to the tracer concentration in the last subsegment of edge *E*_*β*,*jk*_, which is identical to the first subsegment upstream of node *k*. Similar equations as (26) apply to the nodes under the assumption of full porosity within the distributing node volume

$$\begin{array}{cc} \int_{N_{a,k}}{\tilde{\phi }}_{a,k}\frac{\partial {\tilde{c}}_{a,k}}{\partial t}dx & =\Delta f_{a,k}\\ \Delta f_{a,k} & =\{\begin{array}{ccc} & -\left({\tilde{c}}_{AIF}-{\tilde{c}}_{a,k}\right){\tilde{q}}_{a,k} & \;\text{if}\;k\in N_{a}^{R}\;\text{(root}\;\text{terminal,}\;{\tilde{q}}_{a,k}<0)\\ & \sum_{j\in N(N_{a,k})}c^{{}^{\prime }}{\tilde{q}}_{a,jk} & \;\text{if}\;k\in N_{a}^{I}\;\text{(interior}\;\text{node)}\\ & \Delta c{\tilde{q}}_{a,k} & \;\text{if}\;k\in N_{a}^{T}\;\text{(terminal)} \end{array}\\ c^{{}^{\prime }} & =\{\begin{array}{ccc} & {\tilde{c}}_{a,jk,n_{a,jk}} & \;\text{if}\;{\tilde{q}}_{a,jk}>0\;\text{(incoming}\;\text{fluid)}\;\\ & {\tilde{c}}_{a,k} & \;\text{if}\;{\tilde{q}}_{a,jk}<0\;\text{(outgoing}\;\text{fluid)}\; \end{array}\\ \Delta c & =\{\begin{array}{ccc} & {\tilde{c}}_{a,jk,n_{a,jk}}-{\tilde{c}}_{a,k} & \;\text{if}\;q_{a,k}>0\;\text{(terminal}\;\text{is}\;\text{a}\;\text{source)}\;\\ & \int_{\Omega }{\tilde{c}}_{a,k}\eta_{a,k}^{\epsilon }\left(x-x_{k}\right)dx-{\tilde{c}}_{a,k} & \;\text{if}\;q_{a,k}<0\;\text{(terminal}\;\text{is}\;\text{a}\;\text{sink)}\; \end{array}\\ \int_{N_{v,k}}{\tilde{\phi }}_{v,k}\frac{\partial {\tilde{c}}_{v,k}}{\partial t}dx & =\Delta f_{v,k}\\ \Delta f_{v,k} & =\{\begin{array}{ccc} & \left({\tilde{c}}_{v,jk,n_{v,jk}}-{\tilde{c}}_{v,k}\right){\tilde{q}}_{v,k} & \;\text{if}\;k\in \left(N_{v}^{I}\cup N_{v}^{R}\right)\;\text{(root}\;\text{terminal,}\;{\tilde{q}}_{a,k}>0)\\ & \sum_{j\in N(N_{v,k})}c^{{}^{\prime }}{\tilde{q}}_{v,jk} & \;\text{if}\;k\in N_{v}^{I}\;\text{(interior}\;\text{node)}\\ & \Delta c{\tilde{q}}_{v,k} & \;\text{if}\;k\in N_{v}^{T}\;\text{(terminal)} \end{array}\\ c^{{}^{\prime }} & =\{\begin{array}{ccc} & {\tilde{c}}_{v,jk,n_{v,jk}} & \;\text{if}\;{\tilde{q}}_{v,jk}>0\;\text{(incoming}\;\text{fluid)}\;\\ & {\tilde{c}}_{v,k} & \;\text{if}\;{\tilde{q}}_{v,jk}<0\;\text{(outgoing}\;\text{fluid)}\; \end{array}\\ \Delta c & =\{\begin{array}{ccc} & {\tilde{c}}_{v,jk,n_{v,jk}}-{\tilde{c}}_{v,k} & \;\text{if}\;q_{v,k}>0\;\text{(terminal}\;\text{is}\;\text{a}\;\text{source)}\;\\ & \int_{\Omega }{\tilde{c}}_{v,k}\eta_{v,k}^{\epsilon }\left(x-x_{k}\right)dx-{\tilde{c}}_{v,k} & \;\text{if}\;q_{v,k}<0\;\text{(terminal}\;\text{is}\;\text{a}\;\text{sink)}\; \end{array} \end{array}$$ (27)

where *f*_*β*,*k*_ is the incoming/outgoing tracer flux of node *k*. Within the edges, tracer concentrations in each subsegment follows accordingly

$$\begin{array}{ccc} \int_{E_{\beta ,jk,i}}{\tilde{\phi }}_{\beta ,jk,i}\frac{\partial {\tilde{c}}_{\beta ,jk,i}}{\partial t}dx=\left({\tilde{c}}_{\beta ,k}-{\tilde{c}}_{\beta ,jk,i}\right){\tilde{q}}_{\beta ,jk} & & i=1\\ \int_{E_{\beta ,jk,i}}{\tilde{\phi }}_{\beta ,jk,i}\frac{\partial {\tilde{c}}_{\beta ,jk,i}}{\partial t}dx=\left({\tilde{c}}_{\beta ,jk,i-1}-{\tilde{c}}_{\beta ,jk,i}\right){\tilde{q}}_{\beta ,jk} & & i=2,\ldots ,n_{\beta ,jk} \end{array}$$ (28)

for *β* = {*a*, *v*}. Note that the first subsegment relates to the upstream node. The hematocrit factor *Hct* connects blood tracer concentration *c*_*β*,*b*_ with plasma tracer concentration *c*_*β*_ according to

$$\begin{array}{c} c_{\beta ,b}=c_{\beta }\left(1-Hct\right). \end{array}$$ (29)

Tracer can only distribute within the arterial and the venous compartment, and the observable tracer concentration becomes

$$\begin{array}{c} C\left(x,t\right)=(c_{a}\left(x,t\right)\phi_{a}+c_{v}\left(x,t\right)\phi_{v})\left(1-Hct\right). \end{array}$$ (30)

when applying (22), (23), and (29). In the current model, the hematocrit is independent of vessel scale, and therefore only has the role as a global scaling factor of the tracer concentration curves. Eqs (26), (27), (28), and (30) form the model for indicator dilution. Further details on the numerical implementation are shown in Supporting Information.

### Numerical implementation of flow

Integrating (8) over a control volume (voxel) Ω_*i*_ ⊂ Ω and applying the divergence theorem yields

$$\begin{array}{cc} -\int_{\partial \Omega_{i}}\left(\lambda_{a}\nabla p_{a}\right)\cdot ndA & =\sum_{k\in T_{a}^{I}}\int_{\Omega_{i}}Q_{a,k}^{\epsilon }dx-\int_{\Omega_{i}}Pdx\\ -\int_{\partial \Omega_{i}}\left(\lambda_{v}\nabla p_{v}\right)\cdot ndA & =\sum_{k\in T_{v}^{I}}\int_{\Omega_{i}}Q_{v,k}^{\epsilon }dx+\int_{\Omega_{i}}Pdx \end{array}$$ (31)

for the conductivities λ_*β*_ ≔ *k*_*β*_/*μ*, *β* = {*a*, *v*}. The elliptic term of Eq (8) was discretized using finite volume TPFA (two-point flux approximation), leading to a linear relation in the transmissibilities *t*_*ij*_ and pressure difference *p*_*β*,*i*_ − *p*_*β*,*j*_ between a center voxel *x*_*i*_ and an adjacent neighbor *x*_*j*_. TPFA is widely applied in reservoir mechanics, and the reader is referred to [43] for more details. Following TPFA, Eq (31) can be approximated as a linear system

$$\begin{array}{ccc} & \sum_{j\in N_{i}}t_{ij}\left(p_{a,i}-p_{a,j}\right)+\alpha_{i}\left(p_{a,i}-p_{v,i}\right)|\Omega_{i}|-\sum_{k\in T_{a}^{I}}{\tilde{q}}_{a,k}\eta_{a,k}^{\epsilon }\left(x_{i}-x_{k}\right)\left|\Omega_{i}\right|=0 & \\ & {\tilde{q}}_{a,k}=\kappa_{a,jk}\left({\tilde{p}}_{a,j}-{\tilde{p}}_{a,k}\right),\;j\in N\left(N_{a,k}\right)\;\forall \;k\in N_{a}^{I}\;\text{Continuity}\;\text{in}\;\text{flow}\\ & \sum_{j\in N_{i}}t_{ij}\left(p_{v,i}-p_{v,j}\right)-\alpha_{i}\left(p_{a,i}-p_{v,i}\right)|\Omega_{i}|-\sum_{k\in T_{v}^{I}}{\tilde{q}}_{v,k}\eta_{v,k}^{\epsilon }\left(x_{i}-x_{k}\right)\left|\Omega_{i}\right|=0 & \\ & {\tilde{q}}_{v,k}=\kappa_{v,jk}\left({\tilde{p}}_{v,j}-{\tilde{p}}_{v,k}\right),\;j\in N\left(N_{v,k}\right)\;\forall \;k\in N_{v}^{I}\;\text{Continuity}\;\text{in}\;\text{flow} \end{array}$$ (32)

when also applying Eq (20). Further define the voxel neighborship around an interior node $N_{v}\left(N_{\beta ,k}\right)≔\left\{j:\eta_{\beta ,k}^{\epsilon }\left(x_{j}-x_{k}\right)>0,k\in N_{\beta }^{I}\right\}$ including all voxels close to terminal *N*_*β*,*k*_ receiving a nonzero fluid distribution. The network Eqs (11) and (12) are readily discretized, while the condition on pressure continuity Eq (21) becomes

$$\begin{array}{ccc} & {\tilde{q}}_{\beta ,k}=\frac{\gamma_{\beta }}{\mu }({\tilde{p}}_{\beta ,k}-\sum_{j\in N(N_{\beta ,k})}\eta_{\beta ,k}^{\epsilon }\left(x_{j}-x_{k}\right)p_{\beta ,j}\left|\Omega_{j}\right|)\; & \forall \;k\in N_{\beta }^{I}\; \end{array}$$ (33)

A linear system Ax=d was created,

$$A=\left(\begin{array}{cc} \begin{array}{cc} A\left(D_{a}\to D_{a}\right) & A\left(D_{a}\to D_{v}\right)\\ A\left(D_{v}\to D_{a}\right) & A\left(D_{v}\to D_{v}\right) \end{array} & \begin{array}{cc} A\left(D_{a}\to N_{a}\right) & 0\\ 0 & A\left(D_{v}\to N_{v}\right) \end{array}\\ \begin{array}{cc} A\left(N_{a}\to D_{a}\right) & 0\\ 0 & A\left(N_{v}\to D_{v}\right) \end{array} & \begin{array}{cc} A\left(N_{a}\to N_{a}\right) & 0\\ 0 & A\left(N_{v}\to N_{v}\right) \end{array} \end{array}\right)$$ (34)

where *x* is the concatenation of the voxelwise pressure values *p*_*β*,*i*_ and nodal pressure values ${\tilde{p}}_{\beta ,i}$. The argument *D* refers to the Darcy equation in the continuum, and *N* refers to the nodes. The subscript indicates arterial or venous compartment/tree. The arrow indicates interactions, e.g. the submatrix $A(D_{a}\to N_{a})$ contains the interaction between the arterial compartment and arterial-tree nodes. Right hand side *d* depends on Dirichlet boundary conditions on the pressure. The linear system of equations was solved using GMRES [44] with a tolerance of 10^−6^, and a LUP decomposition for preconditioning.

### Numerical implementation of indicator dilution

A first approximation of the forward time step was initially computed from the largest possible time step satisfying the CFL conditions of the Darcy domain, the nodes, and the segments. However, due to the implementation of a backward Euler solver, we were able to use significantly longer time steps, leading to a sequence of time points *t*_*i*_ = *iδt*, *i* = {0, 1, …, *n*} where *δt* was ten times the maximum time step according to the CFL condition. Total number of iterations became *n* = floor(120/*δt*), where 120 is maximum simulation time. Forward simulation of tracer evolution was performed by creating a discrete linear system of equations from (26), (27), (28)

$$\begin{array}{c} c_{i+1}=c_{i}+\delta t\left(Ac_{i}+b_{i}\right),\;i=\left\{2,3,\ldots ,n\right\},\;c_{0}=0 \end{array}$$ (35)

in the variable

$$\begin{array}{c} c_{i}=[\begin{array}{c} c_{D,a},c_{D,v},c_{N,a},c_{N,v},c_{E,a},c_{E,v} \end{array}{]}_{i}^{T} \end{array}$$ (36)

containing the concatenation of discrete variables of tracer concentration at time point *t*_*i*_ in the Darcy domain *c*_*D*,*β*_, the nodes *c*_*N*,*β*_, and the edges *c*_*E*,*β*_, and where B is a block-diagonal matrix

$$ℬ=\left(\begin{array}{ccc} \begin{array}{cc} ℬ(D_{a}\to D_{a}) & ℬ(N_{a}\to D_{a})\\ ℬ(D_{v}\to D_{a}) & ℬ(D_{v}\to D_{v}) \end{array} & \begin{array}{cc} ℬ(D_{a}\to N_{a}) & 0\\ 0 & ℬ(D_{v}\to N_{v}) \end{array} & \begin{array}{cc} 0 & 0\\ 0 & 0 \end{array}\\ \begin{array}{cc} ℬ(N_{a}\to D_{a}) & 0\\ 0 & ℬ(N_{v}\to D_{v}) \end{array} & \begin{array}{cc} ℬ(N_{a}\to N_{a}) & 0\\ 0 & ℬ(N_{v}\to N_{v}) \end{array} & \begin{array}{cc} ℬ(N_{a}\to E_{a}) & 0\\ 0 & ℬ(N_{v}\to E_{v}) \end{array}\\ \begin{array}{cc} 0 & 0\\ & 0 \end{array} & \begin{array}{cc} ℬ(E_{a}\to N_{a}) & 0\\ 0 & ℬ(E_{v}\to N_{v}) \end{array} & \begin{array}{cc} ℬ(E_{a}\to E_{a}) & 0\\ 0 & ℬ(E_{v}\to E_{v}) \end{array} \end{array}\right)$$ (37)

with similar notation as (34), in addition to *E*_(⋅)_ referring to the edges. The constant vector *b*_*i*_ depends on AIF values *c*_AIF,*i*_. A backward Euler updates the concentration at time point *t*_*i*_ according to

$$\begin{array}{c} (I-\delta tA)c_{i+1}=c_{i}+\delta tb_{i} \end{array}$$ (38)

where *I* is the identity matrix. The matrix (I-δtB) is fixed over iterations, and a GMRES solver was used as a solver with a LUP preconditioner with the previous iterate as initial guess in the consecutive time iteration.

### Arterial input function

As arterial input function we used a gamma-variate function

$$\begin{array}{c} {\tilde{c}}_{AIF}\left(t\right)=C_{0}{\left(t-t0\right)}^{A}e^{-(t-t0)/B} \end{array}$$ (39)

with constants *A* = 3, *B* = 1 [45]. Tracer simulation time was 120 s, with a delay *t*_0_ = 7.5 min. All program code was written in MATLAB.

### Sensitivity analysis

We performed a numerical sensitivity analysis to examine how uncertanties in the input parameters are propagating through the model and affecting the output parameters. For a function *y* = *f*(*x*_*i*_) depending on a set of variables *x*_*i*_, *i* = {1, 2, …}, the relative sensitivity coefficient

$$\begin{array}{c} c_{i}^{*}≔\frac{x_{i}}{y}\frac{\partial y}{\partial x_{i}} \end{array}$$ (40)

is a measure of how the input parameter *x*_*i*_ affects the outcome *y*. The derivative was computed around an expected *x*_*i*_ with a 1% variation on *x*_*i*_. We report $c_{i}^{*}$ for the perfusion parameter *α*, the fluid viscosity *μ*, as well as the arterial and venous components of the porosity *ϕ*_*β*_, the permeability *k*_*β*_, and the pressure drop parameter *γ*_*β*_. As investigated output parameters we used the arterial and venous pressures *p*_*a*_ and *p*_*v*_, respectively, the perfusion (P), time to peak (TTP), and the mean transit time (MTT). Time to peak and mean transit time were computed according to standard definitions from tracer kinetic modelling. Time to peak is the average time in seconds to maximum height of the contrast enhancement curves. Mean transit time becomes CBV/CBF, which is equivalent to MTT = (*ϕ*_*a*_ + *ϕ*_*v*_)/*P* in terms of our notation.

## Results

### Numerical simulation of circulation and perfusion in a frog tongue

**Fig 5:**
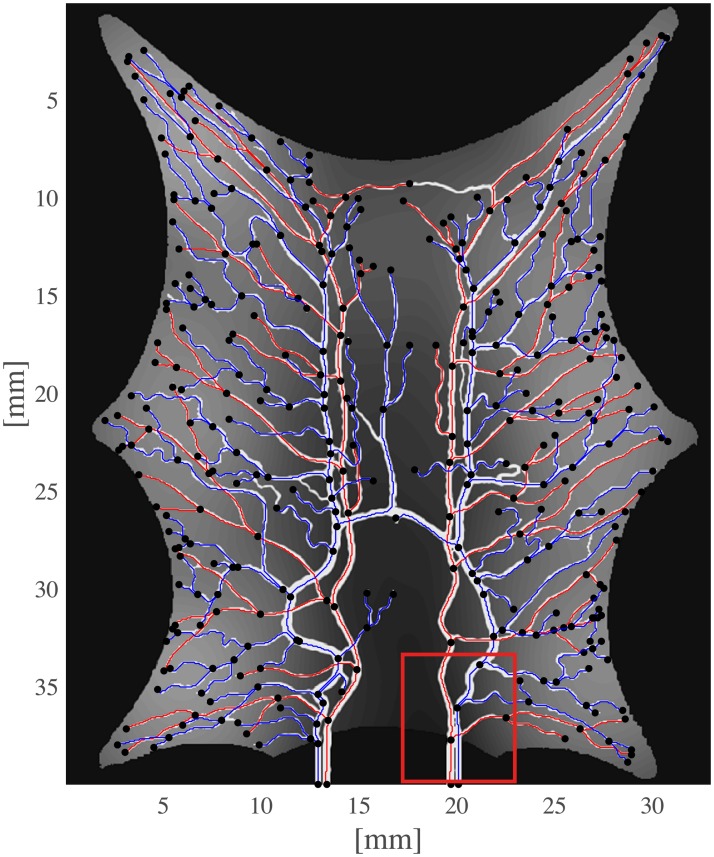
Vascular network of the frog tongue. Node centers are indicated with black dots. The medial axis of the network structure is shown as the set of lines connecting the nodes (red lines: arterial network, blue lines: venous network). The grey area within the tongue tissue indicates the support function *η*^*ϵ*^(*x* − *x*_*k*_) (15) summed up for all terminals. The red rectangle in the lower field is the small FOV used for demonstrating scale invariance.

**Fig 6:**
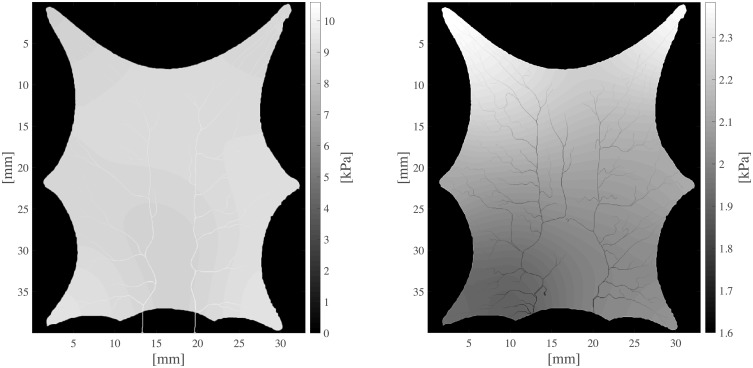
Pressure maps *p*_*a*_ and *p*_*v*_ of arterial (left) and venous compartment (right) of the frog tongue, respectively. Note the different greyscale range between the plots, applied for visualization purposes.

**Fig 7:**
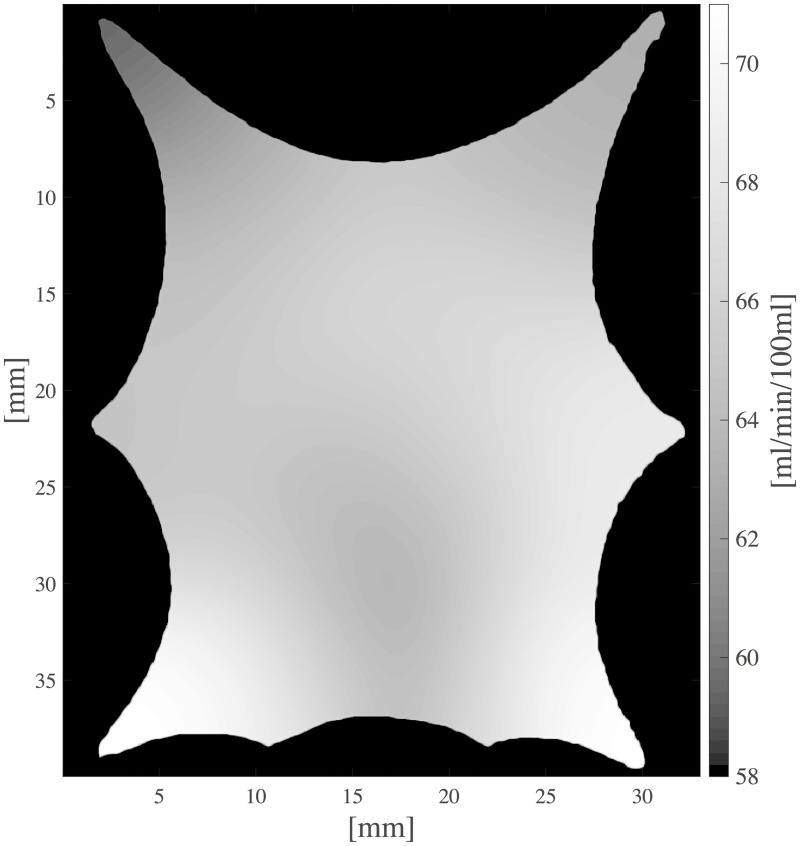
Regional variability of the perfusion *P* [ml/min/100ml] within the frog tongue.

**Fig 8:**
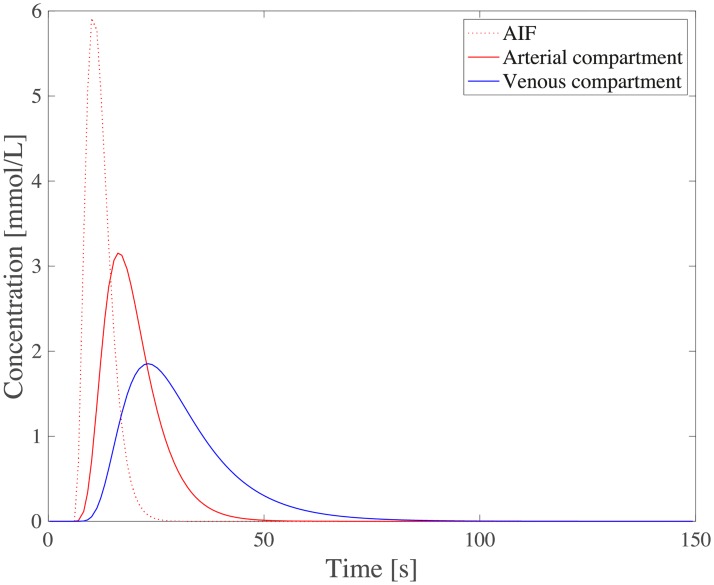
Average fluid tracer concentration [mmol L^−1^] as a function of time for the arterial input function (AIF), and for the arterial and venous compartment of the frog tongue. The average was calculated over the frog tongue.

**Fig 9:**
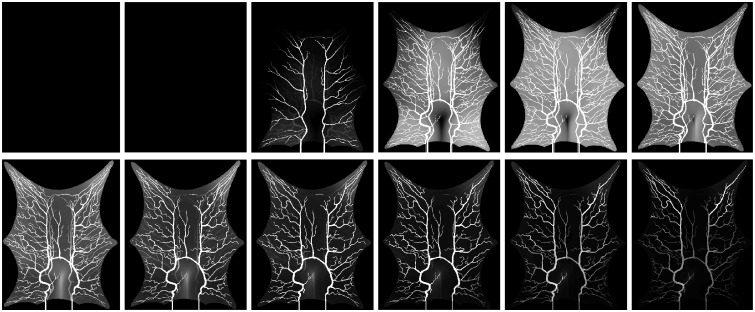
Voxelwise volumetric tracer concentration *C* [mmol L^−1^] (22) as a function of time for the frog tongue. Every five second is shown from left to right and top to bottom, T = {0, 5, …, 60} s.

**Fig 10:**
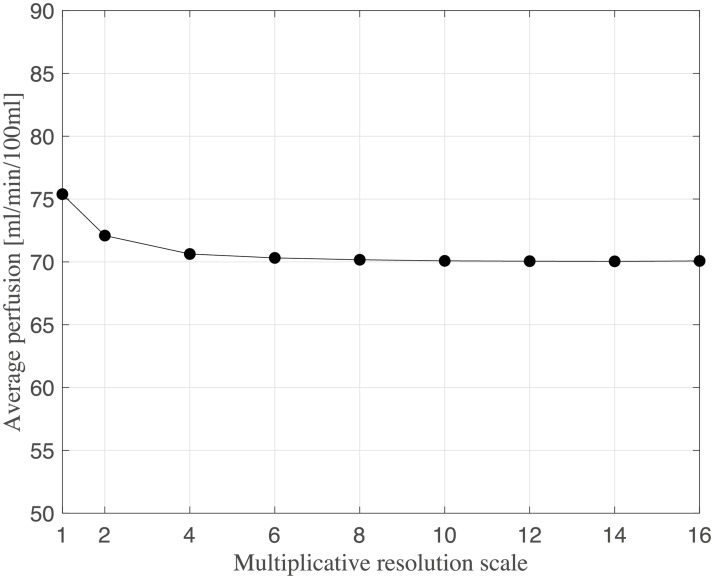
Average brain perfusion computed within the same FOV but under various multiplicative resolution scales. All other simulation parameters were identical across the resolution scales. The average perfusion is converging at higher resolution scales.

The current section accounts for simulation of circulation and perfusion in the frog tongue previously described. The vascular networks were segmented in terms of a binary mask, nodes, edges, and medial axes. In Fig 5 we have aligned these structures with the support function *η*^*ϵ*^(*x* − *x*_*k*_) (15). Simulation parameters used in the numerical simulations are shown in Table 1. Several of the parameters are not accurately known, and literature references were used to find appropriate estimates. The parameters *k*_*β*_ and *ϕ*_*β*_ are field parameters, but were held constant in space within each compartment. The perfusion proportionality factor *α* was held constant everywhere within the frog tongue. Obtained pressure maps of the arterial and venous compartments are depicted in Fig 6. Pressure conditions in the vessel network are completely determined by the node pressure, but for visualization purposes the pressure was approximated along the edges using linear interpolation between connecting nodes. The obtained map of perfusion is shown in Fig 7. For the applied set of parameters an average perfusion of 65 ml/min/100ml was obtained, in the same order as human brain perfusion [50]. Average fluid tracer concentration of the arterial input function, and the arterial and venous compartments are shown in Fig 8. Voxelwise, volumetric tracer concentration *C* [mmol L^−1^] (30) as a function of time is shown in Fig 9. We used a time step Δ*t* = 5 s between each time frame for plotting. In order to demonstrate scale invariance of the algorithm, perfusion was recomputed within a smaller FOV with different resolutions represented by multiplicative resolution scales *S*_*i*_, *i* = {1, 2, 4, 6, 8, 10, 12, 14, 16}. See the red rectangle in Fig 5 for the applied FOV with matrix size *S*_1_ = (100 × 100). The matrix size at scale *i* becomes *S*_*i*_ = (100 × 100)*i*. With except from the FOV, same parameter settings as reported in Table 1 were used for these simulations. Average perfusion for each scale was computed within the frog tongue, and obtained values are shown in Fig 10. For all practical means, perfusion remains constant over the resolution scales.

**Table 1:** Scalar simulation parameters for the frog tongue within various domains. Matrix size was limited by the native input data. Arterial and venous boundary pressure values were applied as Dirichlet boundary conditions to the vascular root terminals *N*^*R*^. The perfusion proportionality factor *α*(*x*) (7) was assigned a constant value everywhere. par. = parameter. Field parameter *α* is only valid for the brain $\Omega_{B}$.

Parameter	Symbol	Unit	Domain of validity				
			Entire domain	Compartment		Vascular network	
				Arterial	Venous	Arterial	Venous
Field-of-view	FOV	mm	(40, 33, 1)				
Matrix size	-	-	(634, 515, 1)				
Voxel size	*h*	mm	(0.063, 0.064, 1)				
Viscosity blood [46]	*μ*_*b*_	Pa ⋅ s	3.00 × 10^−3^				
Support radius (17)	*ϵ*	mm	10.0				
Hematocrit	Hct	-	0.40				
Perfusion par. (7)	*α*	m s kg^−1^	1.00 × 10^−6^				
Permeability [47]	*k*_*β*_	10^−12^ m^2^		1.00	5.00		
Porosity [48]	*ϕ*_*β*_	-		0.05	0.10		
Boundary pressure [49]	*p*_*β*,0_	kPa				10.6	1.60
Pressure drop par. (21)	*γ*_*β*_	10^−12^ m^3^				0.01	0.01

### Whole brain simulation on a MRI-extracted geometry

**Fig 11:**
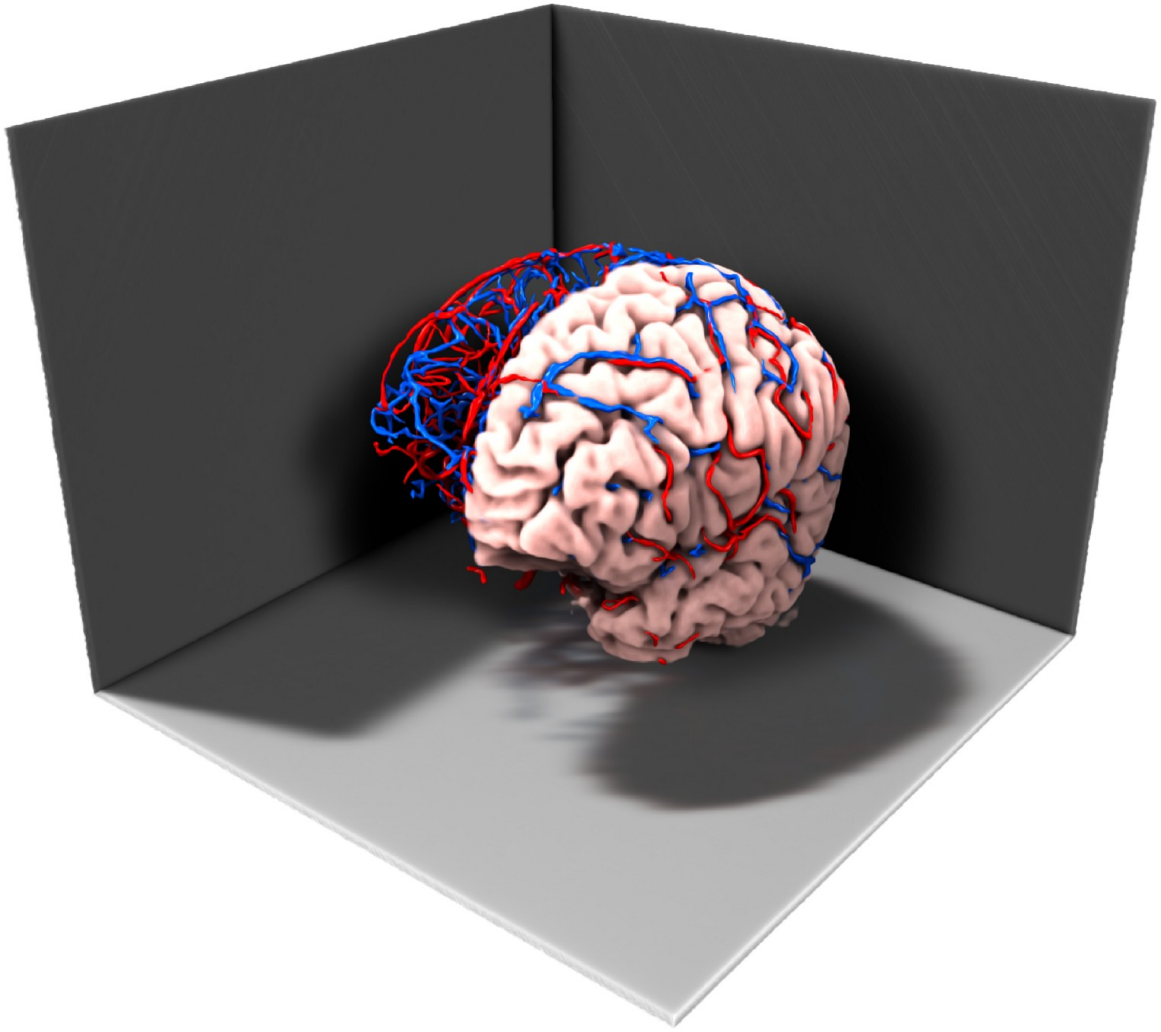
3D volume rendering of the T1-weighted data including the arterial (red) and venous (blue) vessels.

The current section describes numerical simulation of circulation and perfusion in a complete human brain where the geometry, including grey and white matter, as well as the vascular networks were extracted from MRI data. Simulation parameters are shown in Table 2. All figures show the same image slice (no. 180) of the 3D image stack, with except from the 3D rendering in Fig 11.

**Table 2:** Simulation parameters for the human brain geometry within the various sub-domains. Arterial and venous boundary pressures were applied as Dirichlet boundary conditions to the vascular root terminals *N*^*R*^. Permeability and porosity are field parameters, but were assigned constant values within each tissue and compartment. par. = parameter.

Parameter	Symbol	Unit	Domain of validity				
			Entire domain	Compartment		Vascular network	
				Arterial	Venous	Arterial	Venous
Field-of-view	FOV	mm	(170, 220, 153)				
Matrix size	-	-	(346, 448, 311)				
Voxel size	*h*	mm	(0.49, 0.49, 0.49)				
Viscosity blood [46]	*μ*_*b*_	[Pa ⋅ s]	3.00 × 10^−3^				
Support radius (17)	*ϵ*	mm	30.0				
Hematocrit	Hct	-	0.40				
Perfusion par. (7)	*α*	m s kg^−1^	1.00 × 10^−5^				
Permeability [47]	*k*_*β*_	10^−12^ m^2^		12.5	25.0		
Porosity [48, 51]	*ϕ*_*β*_	-		0.05	0.10		
Boundary pressure [49]	*p*_*β*,0_	kPa				13.3	0.66
Pressure drop par. (21)	*γ*_*β*_	10^−12^ m^3^				0.20	0.20

**Fig 12:**
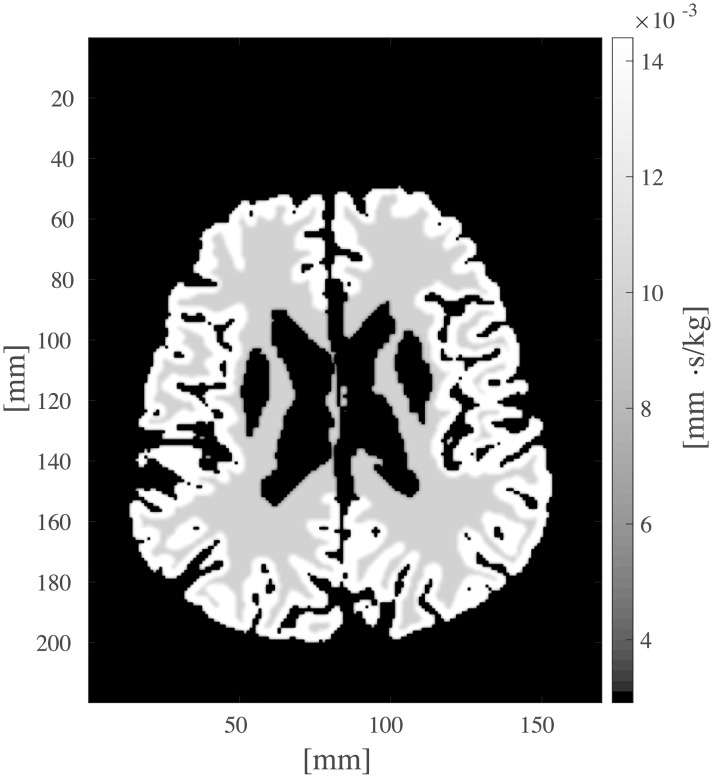
Map of the perfusion proportionality factor *α*(*x*) used as input to the simulations. Higher perfusion was assigned to grey matter than to white matter in order to resemble regional distribution of perfusion within a real human brain.

**Fig 13:**
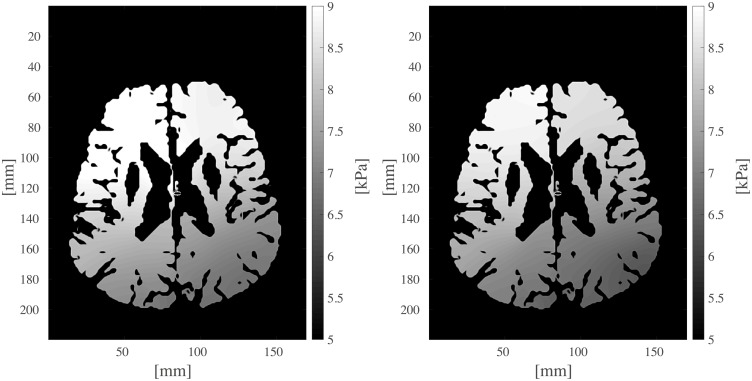
One slice of the calculated pressure maps *p*_*a*_ and *p*_*v*_ of the arterial (left) and venous (right) compartment, respectively.

**Fig 14:**
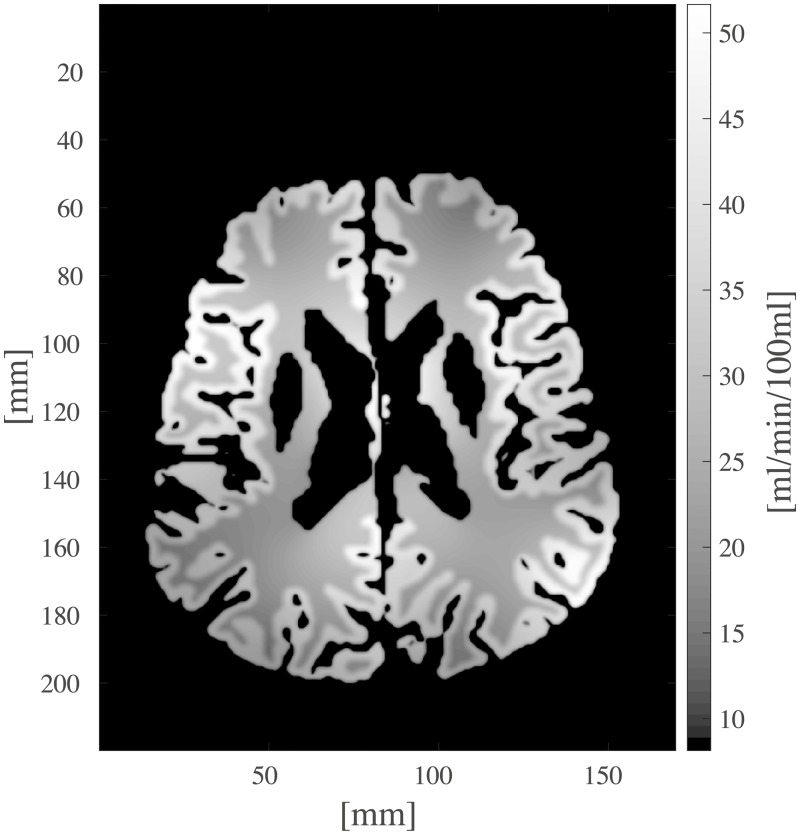
Obtained voxelwise perfusion *P* [ml/min/100ml] (7) for one axial slice.

**Fig 15:**
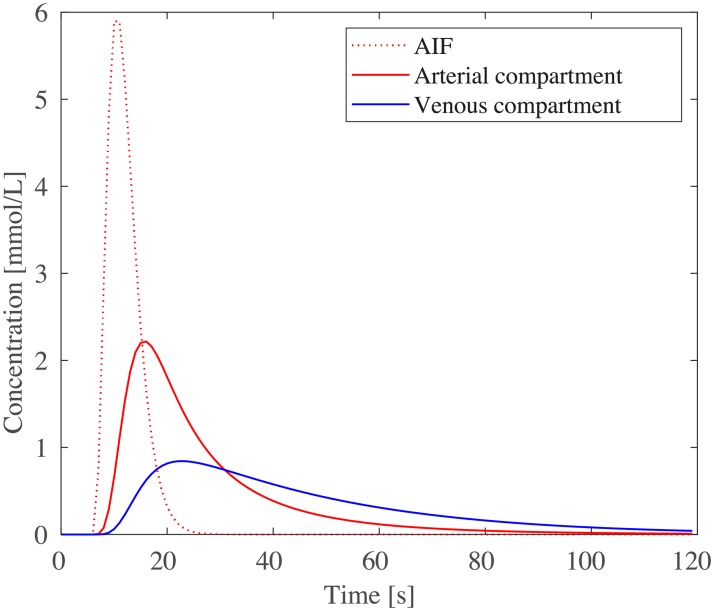
Average tracer concentration time curves within the arterial input function, as well as the arterial and venous compartments of the human brain dataset.

A volume rendering of the T1-weighted input data with superimposed arterial and venous masks is shown in Fig 11 [52]. Vascular permeability *k*_*β*_ and porosity *ϕ*_*β*_ were assigned constant values within grey and white matter for each compartment, according to Table 2. The perfusion proportionality factor *α*(*x*) (7) is plotted in Fig 12 for one axial slice. The grey matter value of *α* was set 1.6 times higher than the white matter value in order to resemble regional distribution of human brain perfusion [42]. The piecewise constant parameter maps of *k*_*β*_(*x*), *ϕ*_*β*_(*x*), and *α*(*x*) were smoothed using a Gaussian convolution with radius 2.5 mm and standard deviation 1.5 mm to impose smoothness in the white matter/grey matter boundary. The Gaussian smoothing is an attempt to simulate partial volume effects in MR, where a voxel situated on the boundary between different tissue will possess properties reflecting both tissue types. Calculated pressure maps of the arterial and venous compartments are shown in Fig 13. The voxelwise map of perfusion for a single axial slice is shown in Fig 14. Obtained values of average perfusion, arterial and venous pressure, time to peak (TTP), and mean transit time (MTT) are reported in Table 3 for the entire brain, as well as for white, and grey matter. The ratio of white matter perfusion to grey matter perfusion is 1.45, not far away from the expected ratio of approximately 1.6. The total number of arterial and venous nodes found in the data set was 335 and 1222, respectively. Spatially averaged tracer concentration-time-curves are shown in Fig 15 for the arterial input function, as well as the arterial and venous compartments. Run-time for the whole brain simulation was 2.5 d on a 32 multicore 2.29 GHz linux server with 355 Mb RAM without use of parallel computing environments.

**Table 3:** Obtained average perfusion $\bar{P}$, arterial ${\bar{p}}_{a}$ and venous ${\bar{p}}_{v}$ pressure, time to peak ($\bar{\text{TTP}}$), and mean transit time ($\bar{\text{MTT}}$) for various brain regions in the whole brain simulation.

Brain region	$\bar{P}$	${\bar{p}}_{a}$	${\bar{p}}_{v}$	$\bar{\text{TTP}}$	$\bar{\text{MTT}}$
Unit	mL/min100/mL	Pa	Pa	s	s
Entire brain	35.78	8.10	7.62	17.03	35.30
White matter	29.20	8.15	7.68	18.01	40.36
Grey matter	42.21	8.06	7.57	17.03	30.36

### Parameter sensitivity analysis

**Fig 16:**
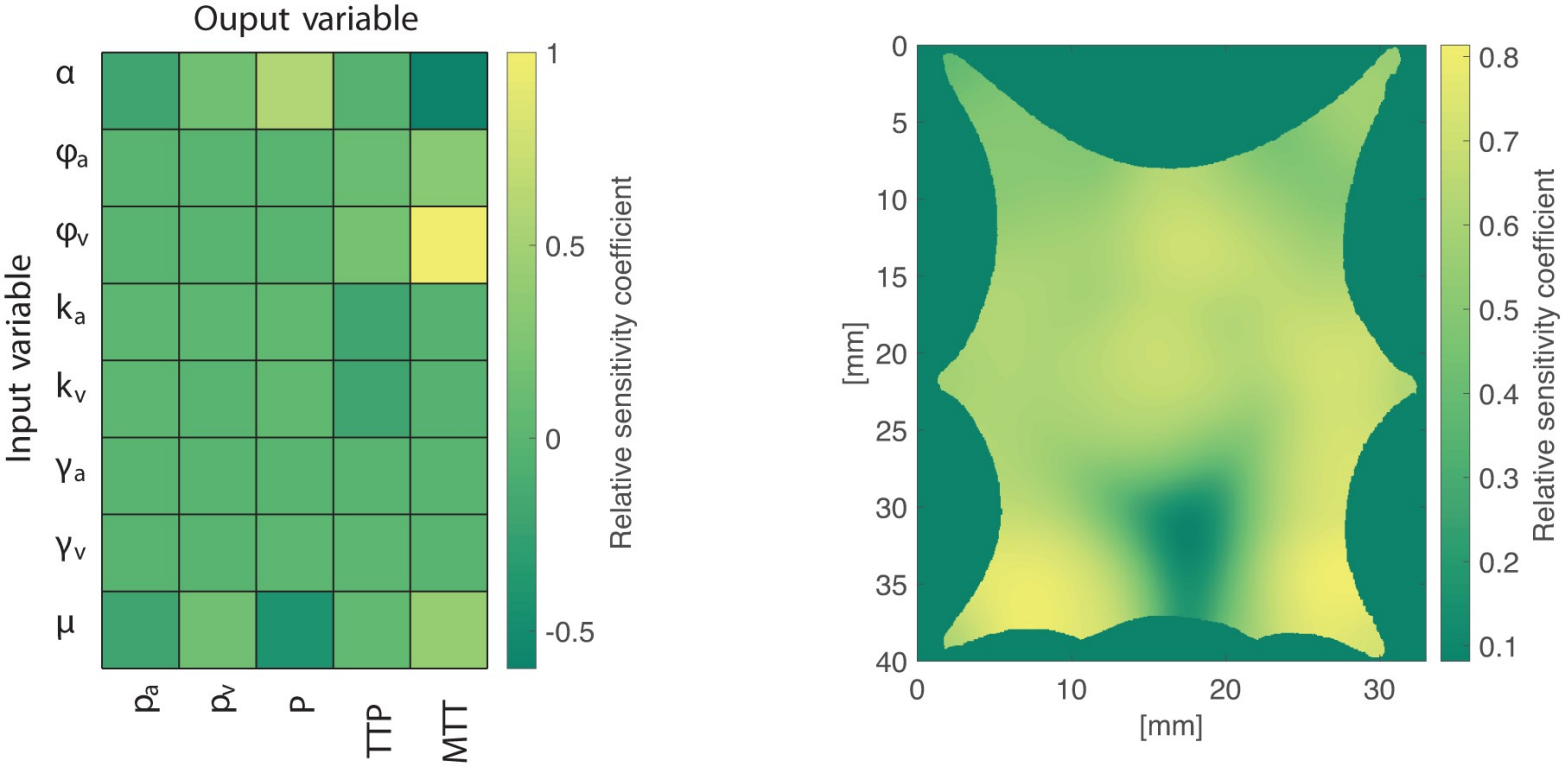
Relative sensitivity coefficients for the perfusion proportionality parameter *α*, porosities *ϕ*_*a*_, *ϕ*_*v*_, vascular permeabilities *k*_*a*_, *k*_*v*_, the pressure drop parameter *γ*_*a*_, *γ*_*v*_, and fluid viscosity *μ* investigated for the compartmental pressures *p*_*a*_, *p*_*v*_, the perfusion *P*, time to peak (TTP), and mean transit time (MTT) for the frog tongue. Brighter values indicate higher sensitivity of the input parameter to the output parameter. Left: Relative sensitivity coefficient averaged over the voxels for the 3D human brain example. Right: Voxelwise, relative sensitivity coefficients from the frog tongue showing the relation between *α* and *P*.

The relative sensitivity coefficient $c_{i}^{*}$ according to (40) was computed for each output variable for the frog tongue and the 3D human brain example. Spatially averaged relative sensitivity coefficients of the human brain example are shown in Fig 16 (left panel), where it is found that the perfusion proportionality parameter *α* has a strong positive relation to the perfusion *P* (grid position (1,3)), and a negative relation to MTT (grid position (1,5)). Venous porosity *ϕ*_*v*_ is strongly positively correlated with MTT (grid position (3,5)). An example of a voxelwise map of $c_{i}^{*}$ for the frog tongue is shown in Fig 16 (right panel), demonstrating a local variability in the coefficients.

## Discussion

A proper mathematical model of circulation and perfusion is essential for simulating the pathway of blood, nutrients, oxygen, and drugs within the brain and other organs. In this respect, a comprehensive simulation tool for entire organs should address certain requirements: (i) It needs to be scale invariant, (ii) it should reflect a clinical understanding of perfusion, and (iii) it should apply to an entire organ. We claim that existing simulation tools are lacking at least one of these requirements. Traditional pharmacokinetic compartment models are useful to describe perfusion within entire organs, but they are inaccurate for voxelwise descriptions [6–9], hence, they are short of (i). On the other hand, numerous simulation studies have described the intertwined processes of angiogenesis, drug delivery and interstitial flow in artificial tumor microvascular networks [12–15]. However, these models are not explicitly expressing perfusion as a clinical parameter, and they have not been developed for the whole brain, hence lacking in (ii) and (iii). In this context, there is a need for precise mathematical models describing perfusion both locally and globally, also requested in [53]. The current study is an attempt to bridge this gap. Our main contribution is a comprehensive, data-driven and scale invariant model for whole brain circulation and perfusion using a mathematically strict definition of perfusion in line with its clinical understanding. Thus, our method is simultaneously addressing (i)-(iii), and it represents a new generation of simulation tools for predicting transport of nutrients, oxygen and drugs in live, human tissue. Simulation of flow in live, human tissue is a challenging task where compartment models with suitable modifications can be transferred across organs and physiology [18]. In this context, our model is generic and can be modified for description of other organs than the brain, as well as pathological conditions. At present, our model assumes no leakage and an intact blood-brain barrier, and the focus for discussion is restricted to brain perfusion in the absence of pathological conditions.

In the current study we demonstrate scale invariance of our algorithm, thus fulfilling (i). This becomes clear in Fig 10, where the average perfusion within a patch convergences towards a fixed number at higher multiplicative scales. In this respect, we have shown that our method can be used to retrieve or simulate perfusion in isolated patches of various scale and resolution given appropriate boundary conditions. Practically speaking, the average perfusion over a ROI is by all practical means independent of discretization level, a property lacking for traditional compartment models of perfusion. With respect to (ii) above, our implementation is in line with previous work where it was suggested to define perfusion as the transition of blood from the arterial to the venous side in a 2C model [16–19].

An important novelty of our work is the data-driven whole brain geometry applied in the simulations, satisfying (iii) above. Geometry largely affects simulation results by imposing no-flow conditions between the brain and non-brain regions. Our approach to generate the whole brain geometry utilizes advanced MR acquisitions to provide a mask of the brain, and also spatially connected vascular networks in 3D for the arteries and veins. The visibility of the brain mask and the vascular networks is limited by the imaging voxel resolution. A strong argument in favor of whole brain simulation in contrast to simulating on smaller patches only is the application of simpler no-flow boundary conditions around the organ. Also, Dirichlet boundary conditions are effectively applied at the arterial inlet and the venous outlet in the larger vessels. On the contrary, simulating small patches requires unknown and complex boundary conditions along all boundaries with permeating flow.

Our simulation results demonstrate a somewhat higher mean transit time [54], and lower perfusion than expected [55] (i.e. Table 3), although the obtained ratio of grey matter perfusion to white matter perfusion was reasonable. These deviations may be due to erroneous parameter settings, as they must be determined by trial and error. This is also supported by the sensitivity analysis demonstrating a high variability in the sensitivity of input parameters within the investigated parameter range to the output fields (see Fig 16). One useful application of our model is the estimation of local model parameters of porosity, permeability and *α* in the framework of inverse modelling. In these approaches, a tracer kinetic model is fitted to concentration time curves subsequent to a bolus injection of a contrast agent [56]. Common to both forward simulation and inverse modelling tasks is the need for accurate forward kinetic models ensuring conservation of mass. In addition, flow models providing conservation of fluid momentum are applied in more comprehensive models, e.g. ensemble Kalman filters [57]. An initial investigation on estimating parameters of a forward model utilizing a multi-compartment model to estimate perfusion is presented in [58]. The approach presented there is based on the ensemble Kalman filter which is a promising candidate for parameter estimation also in our setting. The ensemble Kalman filter is used for parameter estimation of many large scale problems within geoscience (see e.g. [58]).

A commonly debated challenge of vascular flow simulations is how to connect 1D tubular flow in the vessel network with 3D Darcy flow in the continuum [22, 59, 60]. It is well-known that the numerical accuracy of elliptic PDEs with Dirac source terms is below optimal order estimates since the solution is not in *H*^1^. Several solutions to the problem of numerical instability have been proposed, e.g. modification of the source term itself by smoothing kernels, by locally refined meshes [61], or by quasi-uniform meshes [62]. Our suggested solution circumvents the numerical challenges by modifying the underlying mathematical model of the vascular network, thus becoming compliant with a numerically stable approximation to the PDE. As described earlier, we define a local support function *η*^*ϵ*^ where the flow is distributed from the 1D network into the 3D brain tissue, in this sense avoiding the singularity and also adopting to the physiological circumstances of distributing micro-flow below imaging resolution. The support function ensures fluid mass balance. Furthermore, it assures that a majority of the fluid is distributed close to the terminals rather than further away, while at the same time adopting to local, complex geometrical shapes. Outside a strict threshold *ϵ* no fluid is allocated. For the venous network, the opposite process takes place with fluid uptake instead of delivery within the non-zero support function. The non-local distribution of fluid eliminates the singularities from the mathematical model, at the cost of a (slight) non-locality in the equation. From an abstract perspective, this mimics the situation of modelling fracture propagation in elasticity, where peridynamics is a model that incorporates physically motivated non-local terms [63].

A work of particular relevance is the model in Peyrounette et al. [53]. The authors provide a consistent coupling of the node terminals with the continuum following an analytical approximation of Darcy’s law. In contrast to our model, they require pointwise consistency of pressure drop in adjacent voxels, while we demand consistency in average pressure drop. Also, the authors are not focusing on perfusion as a clinical model parameter, and a whole brain simulation on a data-driven geometry is a task they foresee in future work.

Another approach for dealing with the 1D-3D coupling problem is the simulation of branched mesoscale vessel trees below imaging resolution according to a set of growth rules related to bifurcation angles, vessel radii and length [64]. To the best of our knowledge, whole brain flow simulation on these networks coupled with data-driven macro-scale vessels has not been carried out yet. To this point, we claim that such growth of vessels deprived from attracting forces is mathematically redundant to achieve the goal of a smooth distribution of flow, which is equally well obtained by our proposed method of a smooth support function.

Since we are applying porous media-like flow in the capillaries we are not depending on segmenting single vessels down to the capillary scale. On the macro-scale, we assume the knowledge of vascular networks down to imaging resolution. Hence, there exists an intermediate scale of approximately 50-500 μm with unknown vessel architecture associated with medium-sized arterioles and venules, requiring a novel solution for representing the flow. One proposed solution is the coupling of a 1D vascular network with a hierarchical Darcy flow model [65, 66]. A noticeable challenge following this approach is determination of model parameters for each hierarchical state. Moreover, flow in increasingly larger arterioles does not sufficiently well adhere to classical Darcy flow. Our support function is a novel approach for simultaneously dealing with the 1D-3D coupling problem as well as with the transport of fluid on an invisible, fine-scale network of medium sized arterioles and venules.

A common challenge with the implementation of forward simulation tools is the assignment of accurate parameter settings, which is also a limitation in our work. We tried to accomplish this task by using literature values whenever possible. Another open question is the method’s sensitivity to missing vessel segments below the resolution scale. Clearly, macrostructural geometry is strongly influencing the flow pattern, and even higher resolution data than we apply in our work are expected to produce simulation results with certain differences. However, this is future work of high relevance that should be systematically investigated over a range of admissible resolution scales.

We emphasize that simulation on a full brain geometry is highly challenging from a computational perspective [53, 67]. Indeed, our discrete representation of the whole brain contains 324 arterial segments, 1212 venous segments, and 13.9 millions active voxels on which Darcy’s law is discretized. To our knowledge, no comparable simulations have been conducted at this scale on real data.

In conclusion, we have implemented a scale invariant, whole brain flow model in accordance with a clinical understanding of perfusion. To the best of our knowledge, none of the existing approaches possess these essential properties. Hence, our model represents a new generation of scale invariant simulation tools for brain perfusion, with a wide range of possible applications related to forward simulations as well as to inverse modelling tasks in contrast enhanced imaging. From a clinical point of view, such generic and scale-invariant simulation tools have a large potential to improve the accuracy of postprocessing tools used in dynamic imaging methods. They can also be highly useful for a better understanding of drug delivery and hence treatment efficacy as well as for preoperative planning stages, with a possibly large impact on daily health-care.

## Supporting information

S1 Video 3D rendering of arteries, veins, and brain tissue. Geometric domains for the 3D whole brain simulation including arteries (red vessels), veins (blue vessel), and brain tissue (bright, curved surface). In the simulation, arterial blood moves in on the arterial side, it propagates through capillaries in the brain tissue, and moves out on the other side via the veins.
(MP4)

S1 Data Tissue mask frog. (GZ)

S2 Data Arterial mask frog. (GZ)

S3 Data Venous mask frog. (GZ)

S4 Data White matter mask human brain. (GZ)

S5 Data Grey matter mask human brain. (GZ)

S6 Data Arterial mask human brain. (GZ)

S7 Data Venous mask human brain. (GZ)
